# Plasmin and plasminogen prevent sepsis severity by reducing neutrophil extracellular traps and systemic inflammation

**DOI:** 10.1172/jci.insight.166044

**Published:** 2023-04-24

**Authors:** Juliana P. Vago, Isabella Zaidan, Luiza O. Perucci, Larissa Froede Brito, Lívia C.R. Teixeira, Camila Meirelles Souza Silva, Thaís C. Miranda, Eliza M. Melo, Alexandre S. Bruno, Celso Martins Queiroz-Junior, Michelle A. Sugimoto, Luciana P. Tavares, Laís C. Grossi, Isabela N. Borges, Ayda Henriques Schneider, Nagyung Baik, Ayda H. Schneider, André Talvani, Raphael G. Ferreira, José C. Alves-Filho, Vandack Nobre, Mauro M. Teixeira, Robert J. Parmer, Lindsey A. Miles, Lirlândia P. Sousa

**Affiliations:** 1 Signaling in Inflammation Laboratory, Department of Clinical and Toxicological Analysis, Faculty of Pharmacy, and; 2 Department of Morphology, Institute of Biological Sciences, Universidade Federal de Minas Gerais, Belo Horizonte, Brazil.; 3 Department of Molecular Medicine, The Scripps Research Institute, La Jolla, California, USA.; 4 Department of Biological Sciences, Universidade Federal de Ouro Preto, Ouro Preto, Brazil.; 5 Department of Pharmacology, Faculdade de Medicina de Ribeirão Preto, Universidade de São Paulo, Ribeirão Preto, Brazil.; 6 Department of Biochemistry and Immunology, Institute of Biological Sciences, Universidade Federal de Minas Gerais, Belo Horizonte, Brazil.; 7 Department of Pharmacology, Institute of Biological Sciences, Universidade Federal de Minas Gerais, Belo Horizonte, Brazil.; 8 Hospital of Sciences, Universidade Federal de Minas Gerais, Belo Horizonte, Brazil.; 9 Department of Medicine, Veterans Administration San Diego Healthcare System and University of California, San Diego, California, USA.

**Keywords:** Infectious disease, Bacterial infections, Plasmin

## Abstract

Sepsis is a lethal syndrome characterized by systemic inflammation and abnormal coagulation. Despite therapeutic advances, sepsis mortality remains substantially high. Herein, we investigated the role of the plasminogen/plasmin (Plg/Pla) system during sepsis. Plasma levels of Plg were significantly lower in mice subjected to severe compared with nonsevere sepsis, whereas systemic levels of IL-6, a marker of sepsis severity, were higher in severe sepsis. Plg levels correlated negatively with IL-6 in both septic mice and patients, whereas plasminogen activator inhibitor-1 levels correlated positively with IL-6. Plg deficiency render mice susceptible to nonsevere sepsis induced by cecal ligation and puncture (CLP), resulting in greater numbers of neutrophils and M1 macrophages, liver fibrin(ogen) deposition, lower efferocytosis, and increased IL-6 and neutrophil extracellular trap (NET) release associated with organ damage. Conversely, inflammatory features, fibrin(ogen), and organ damage were substantially reduced, and efferocytosis was increased by exogenous Pla given during CLP- and LPS-induced endotoxemia. Plg or Pla protected mice from sepsis-induced lethality and enhanced the protective effect of antibiotics. Mechanistically, Plg/Pla–afforded protection was associated with regulation of NET release, requiring Pla-protease activity and lysine binding sites. Plg/Pla are important host-protective players during sepsis, controlling local and systemic inflammation and collateral organ damage.

## Graphical abstract

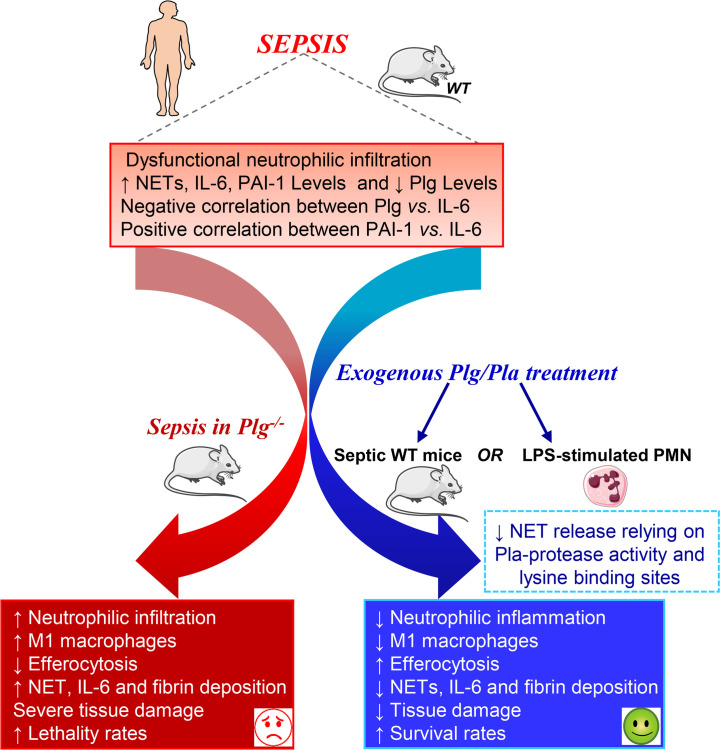

## Introduction

Sepsis is the major cause of death among critically ill patients, contributing to 1 in 3 deaths of hospitalized patients (1). Sepsis commonly results in tissue hypoperfusion, coagulation imbalance, and multiorgan damage linked to intense local and systemic inflammation (2, 3). The intimate connection between inflammation and coagulation in the pathogenesis of several diseases has been increasingly demonstrated (4), whereby inflammation promotes activation of coagulation, and coagulation also markedly contributes to inflammatory responses. This interdependence is clearly evidenced during severe diseases such as COVID-19 and sepsis, in which overwhelming systemic inflammation is associated with hypercoagulation responses (5). Indeed, approximately one-third of all sepsis cases lead to disseminated intravascular coagulation (6, 7), an event intimately connected to death. Therefore, promoting fibrinolysis and controlling overexuberant inflammation to manage thrombosis are emerging therapeutic strategies for the treatment of thromboinflammatory diseases (8–11).

The fibrinolytic system consists of plasminogen (Plg), the zymogen of the serine protease plasmin (Pla), that is synthesized in the liver and present at a concentration of 2 μM in plasma and extracellular fluids (12). Proteolytic activation of Plg to Pla is a mechanism used extensively for degradation of fibrin clots and also for degradation of extracellular matrices and tissue remodeling that results in recruitment of inflammatory cells (12). Activation of Plg to Pla is carried out by tissue plasminogen activator (t-PA) and urokinase-type Plg activator (uPA) (13, 14). The system is negatively regulated by inhibitors of Plg activators (PAI-1 and PAI-2), and by α_2_-antiplasmin (15, 16).

Procoagulant and fibrinolytic components have been proposed to play important roles in controlling inflammation and infection (17). Although Pla activity is increased at the onset of sepsis, increased PAI-1 levels lead to inhibition of fibrinolysis, triggering an imbalance between procoagulant and fibrinolytic systems, which can result in disseminated intravascular coagulation (18). In this regard, recent therapeutic attempts to control the coagulopathy of sepsis have shown promising results, including modulation of inflammation (8, 19). For instance, dabigatran, a potent thrombin inhibitor, promotes the resolution of sepsis through the production of proresolving mediators, which control inflammation resolution (8). Despite the classical view of the fibrinolytic system as an inducer of proinflammatory pathways (12), emerging evidence has illuminated the role of fibrinolysis during the resolution of inflammatory responses (20). Recent studies by our group (21–23) and others (24–27) have revealed anti-inflammatory and proresolving features of the Plg/Pla system through decreasing the production of inflammatory mediators (22, 27), reprogramming M1 to M2 macrophages (21, 22), inducing the production of the proresolving mediator Annexin A1 (21), increasing neutrophil apoptosis (21) and efferocytosis (21, 22, 24), and promoting the nonphlogistic recruitment of mononuclear cells (21, 23).

Thus, in light of the role of the Plg/Pla system in fibrinolysis (14, 20) and its novel functions as a modulator of inflammation (20, 28–31), here we investigated the role of the Plg/Pla system during sepsis, in which systemic inflammation and disseminated intravascular coagulation are major features.

## Results

### Reduced systemic Plg levels are associated with sepsis severity and correlate negatively with IL-6 levels.

**Figure 1:**
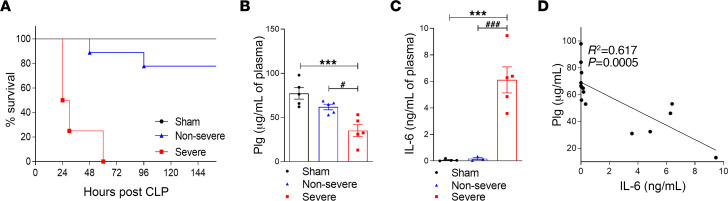
Evaluation of the survival rates and levels of Plg and IL-6 in plasma of mice after severe and nonsevere sepsis. C57BL/6J mice were subjected to severe (18G needle) and nonsevere (30G needle) CLP. (**A**) The survival rates (*n* = 6 mice) were monitored for 6 days. (**B** and **C**) The levels of Plg (**B**) and IL-6 (**C**) were measured in plasma by ELISA 12 hours after CLP. (**D**) The correlation between plasma Plg and IL-6 levels was evaluated by Pearson’s coefficients. Results are shown as the mean ± SEM of at least 5 mice per group. The experiments were performed 3 times with similar results. ***P < 0.001 when comparing sham with severe CLP groups. ^#^*P* < 0.05 or ^###^*P* < 0.001 when comparing severe and nonsevere sepsis groups (1-way ANOVA with post hoc Newman-Keuls).

Plg is a zymogen produced and released by the liver to the bloodstream. After tissue injury or infection, increased levels of Pla are observed, due to tissue plasminogen activator and uPA activation and Plg conversion to Pla (18, 32). Thus, to evaluate whether Plg levels would be altered during sepsis induction and severity, severe and nonsevere models of sepsis induced in mice by cecal ligation and puncture (CLP) were established. The severe model of CLP-induced sepsis led to 100% death within the first 60 hours (Figure 1A), whereas in the nonsevere model, the survival rate was 89% (Figure 1A). Accordingly, sepsis severity was associated with intense leukocyte infiltration into the peritoneal cavity, with neutrophils present at higher numbers in severe compared with nonsevere sepsis (Supplemental Figure 1; supplemental material available online with this article; https://doi.org/10.1172/jci.insight.166044DS1). Interestingly, the plasma levels of Plg in mice subjected to severe sepsis were lower when compared with both sham and nonsevere sepsis groups (Figure 1B). The levels of the cytokine IL-6, considered a marker for sepsis severity, were higher in severe sepsis relative to sham or nonsevere disease (Figure 1C), and a negative correlation between Plg and IL-6 levels during sepsis was observed (Figure 1D). These data suggest that reduction of plasma levels of Plg seen in severe sepsis is associated with disease severity and progression.

### Plg negatively correlates with systemic IL-6 levels in a cohort of septic patients and is reduced in patients with septic shock.

**Figure 2:**
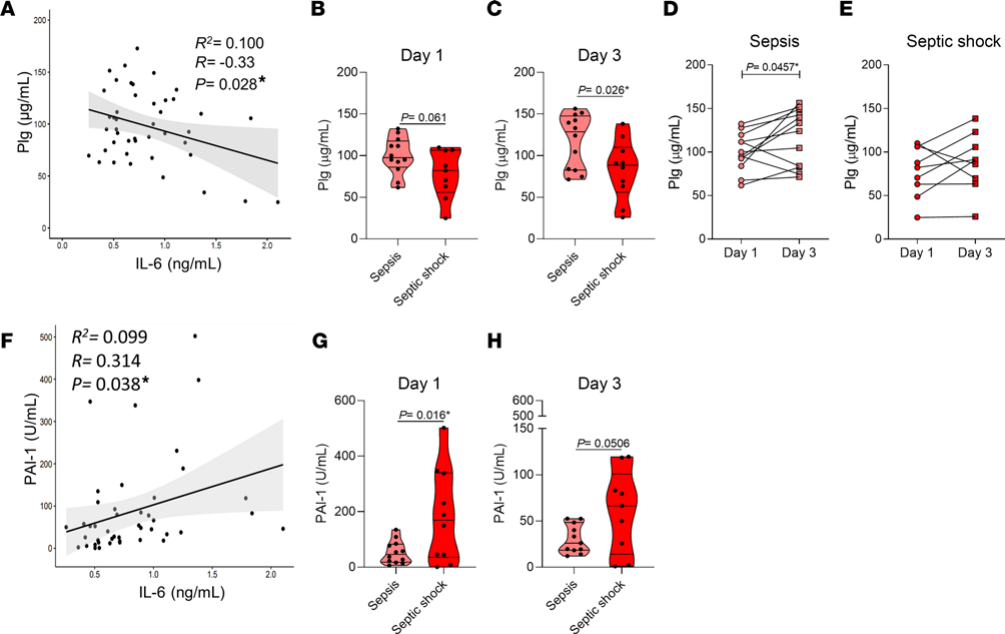
Assessment of Plg levels and PAI-1 in serum of patients with sepsis and septic shock. Blood samples of patients with sepsis were centrifuged and serum levels of Plg, IL-6, and PAI-1 were measured by ELISA. (**A** and **F**) The association between these analytes was evaluated by correlation (Pearson’s coefficient, *R*) and regression analyses (*R*^2^). (**B**, **C**, **G**, and **H**) Plg and PAI-1 levels in patients with sepsis (*n* = 12) and septic shock (*n* = 10) were evaluated on day 1 (**B** and **G**) and day 3 (**C** and **H**) by unpaired 2-tailed Student’s *t* tests. (**D** and **E**) Sequential behavior of Plg levels on days 1 and 3 was evaluated in patients with sepsis (**D**) and patients with septic shock (**E**) by paired 2-tailed Student’s *t* test. *P < 0.05. Outliers were removed from the graphs when detected. Of note, Plg levels in patients with sepsis were measured in serum. Plg levels in both plasma and serum are similar, with serum displaying 16% less Plg than plasma, as previously described (75). Nonetheless, we found consistently high levels of Plg in serum samples from patients with sepsis in our study.

To further investigate the association of Plg levels and sepsis outcome, and whether the evidence from the sepsis mouse model could be translated to humans, samples from patients with sepsis were analyzed. Demographic and clinical parameters of patients with sepsis and septic shock are presented in Supplemental Table 1. Similar to the data obtained from septic mice (Figure 1D), there was a negative correlation between Plg and IL-6 levels in patients with sepsis (Figure 2A). Plg levels were lower in patients with septic shock compared with patients with sepsis, with a trend for reduction (*P* = 0.061) noted on the day of inclusion in the study (Figure 2B) that was significantly different on day 3 (Figure 2C). Notably, levels of Plg increased in patients with sepsis on day 3 compared with day 1 (Figure 2D), whereas no significant differences were observed between these 2 time points in patients with septic shock (Figure 2E). A positive correlation between plasminogen activator inhibitor type-1 (PAI-1) and IL-6 levels was observed (Figure 2F). The levels of PAI-1 have been shown to be positively associated with sepsis severity (33). Indeed, robust PAI-1 activity was observed in patients with septic shock compared with activity in patients with sepsis on the day of patient inclusion (Figure 2G). And although PAI-1 levels dropped at day 3 of hospitalization, there was a trend toward PAI-1 levels remaining high in patients with septic shock (Figure 2H). Patients with septic shock had significantly increased levels of circulating leukocytes and neutrophils, C-reactive protein (CRP), and lactate (Supplemental Table 1). Correlations were negative between Plg levels and CRP and positive between Plg levels and platelets (Supplemental Table 2).

### Plg-deficient mice are more susceptible to CLP-induced sepsis, with intense inflammation and increased lethality rate.

**Figure 3:**
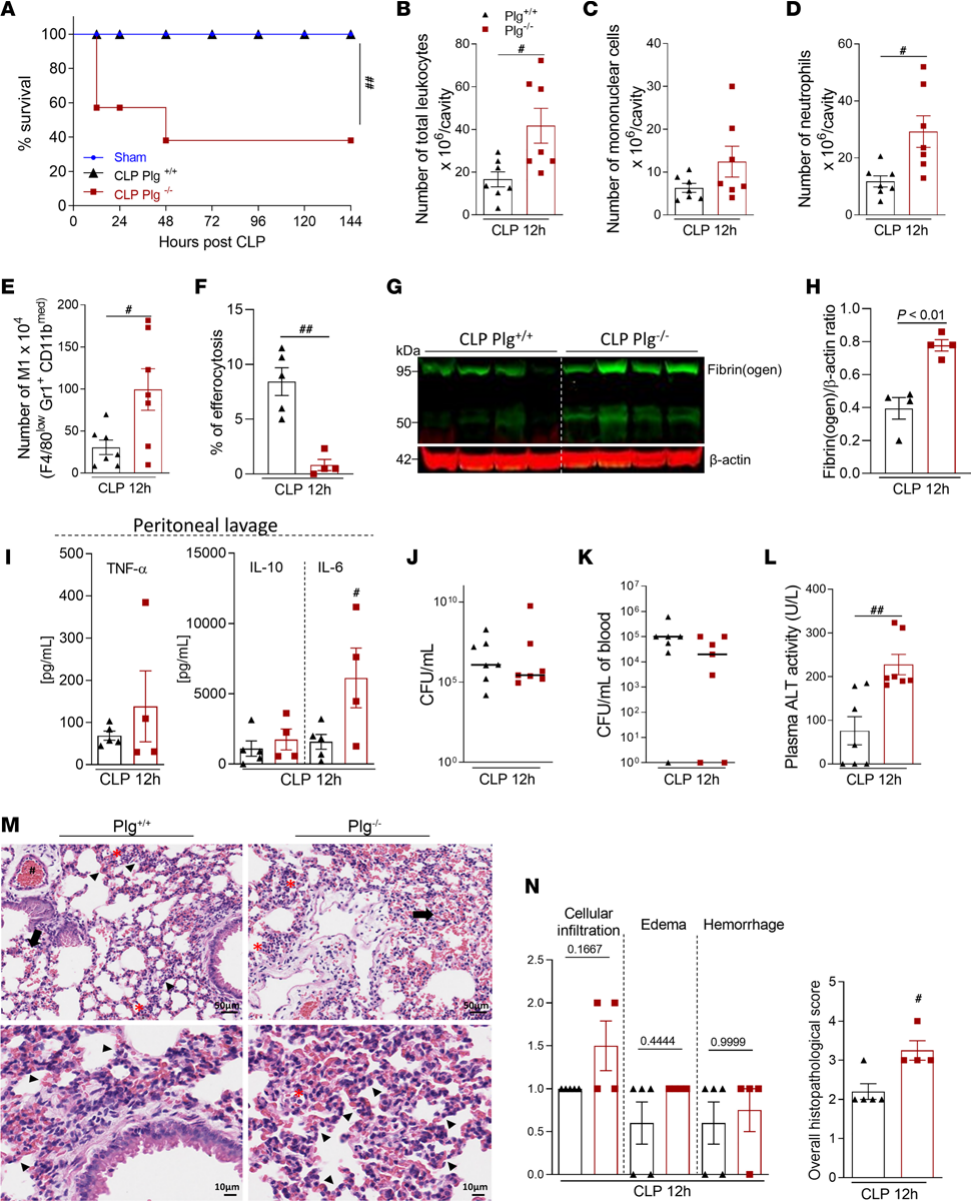
Assessment of survival rates and inflammatory parameters in Plg^–/–^ mice and their WT littermates during nonsevere sepsis. (**A**) Plg^+/+^ and Plg^–/–^ mice were subjected to nonsevere (30G needle) CLP. The survival rates (*n* = 6 mice) were monitored for 6 days. In another set of experiments (*n* = 4–7), cells present in the peritoneal cavity were harvested 12 hours after CLP. (**B–D**) The number of total cells (**B**), mononuclear cells (**C**), and neutrophils (**D**) were evaluated by counting cytospin slides stained with May–Grünwald–Giemsa. (**E**) The number of M1 (F4/80^low^ GR1^+^ CD11b^med^) macrophages was determined by flow cytometry. (**F**) The percentage of efferocytosis was determined by morphological counting of cytospin slides treated with May–Grünwald–Giemsa stain. (**G**) Expression of fibrinogen in the liver was determined by Western blotting with anti–β-actin used as the loading control. (**H**) Densitometric analysis from Western blotting gels is also represented. (**I**) The levels of TNF, IL-10, and IL-6 were quantified by ELISA in cell-free peritoneal lavages. (**J** and **K**) The peritoneal fluid (**J**) and blood (**K**) samples were plated in brain–heart infusion medium for the analysis of bacterial load. (**L**) ALT activity was measured from plasma samples. (**M**) Representative slides of H&E-stained lungs of Plg^+/+^ and Plg^–/–^ mice are shown. *Bottom row:* Higher-magnification images (scale bar: 10 μm) of the micrographs in the *Upper row* (scale bar: 50 μm). (**N**) Histopathological score (maximum score = 5) evaluated focal hemorrhage (arrow), edema (arrowhead), vascular hyperemia (#), and inflammatory infiltrate (*). Results are shown as the mean ± SEM or median of 4–7 mice per group. The experiments were performed 2 times with similar results. ^#^*P* < 0.05 and ^##^*P* < 0.01 when comparing Plg^+/+^ and Plg^–/–^ mice by log-rank test (survival curves), unpaired 2-tailed Student’s *t* test or Mann-Whitney *U* test. Outliers were removed from the graphs when detected.

Because our results show an association between reduced Plg levels and sepsis severity, we next questioned whether Plg deficiency (Plg^–/–^) would influence the progression of nonsevere sepsis. Interestingly, although nonsevere sepsis did not cause any lethality in this cohort of WT (Plg^+/+^) mice (100% survival), a high proportion (62%) of Plg^–/–^ mice succumbed to disease (Figure 3A). Numbers of circulating leukocytes, especially neutrophils, were higher in Plg^–/–^ compared with Plg^+/+^ mice (Supplemental Table 3). Similarly, higher numbers of leukocytes (Figure 3B), predominantly neutrophils (Figure 3D), were present in the peritoneal cavities of Plg^–/–^ mice compared with Plg^+/+^ littermates. At this time of ongoing inflammation, the number of mononuclear cells in the cavity were not significantly different when comparing *Plg^–/–^* and *Plg^+/+^* mice (Figure 3C), but higher numbers of inflammatory M1 macrophages were observed in Plg^–/–^ mice (Figure 3E). Occurrence of neutrophil apoptosis (Supplemental Figure 2) and their clearance by macrophages (efferocytosis) (Figure 3F) was lower in Plg^–/–^ mice subjected to sepsis. Although we found similar plasma fibrinogen levels between the genotypes 12 hours after CLP (CLP-Plg^+/+^, 587.8 ± 87 mg/dL; CLP-Plg^–/–^, 974.4 ± 232 mg/dL; data are presented as mean ± SEM; *n* = 5 mice/group; *P* = 0.158), Plg^–/–^ mice displayed a greater amount of fibrin(ogen) deposition in the liver when compared with Plg^+/+^ (Figure 3, G and H). Importantly, peritoneal IL-6 levels were higher in Plg^–/–^ mice than in their Plg^+/+^ littermates, with no significant changes in levels of TNF and IL-10 (Figure 3I). No differences were found in bacterial counts from peritoneal fluid (Figure 3J) or blood (Figure 3K). However, levels of alanine transaminase (ALT), a marker of liver damage, were increased in Plg^–/–^ mice (Figure 3L). In addition, histological analysis of lung tissue showed increased overall histopathological scores in Plg^–/–^ mice, characterized by increased cellular infiltration, edema, and hemorrhage (Figure 3, M and N). These results suggest that Plg deficiency leads to increased inflammation, defective clearance of neutrophils, tissue damage, and an overall worse outcome from sepsis.

### Exogenous Pla administration reduces local and systemic inflammation, tissue damage, bacteremia, and lethality rates in severe sepsis.

**Figure 4:**
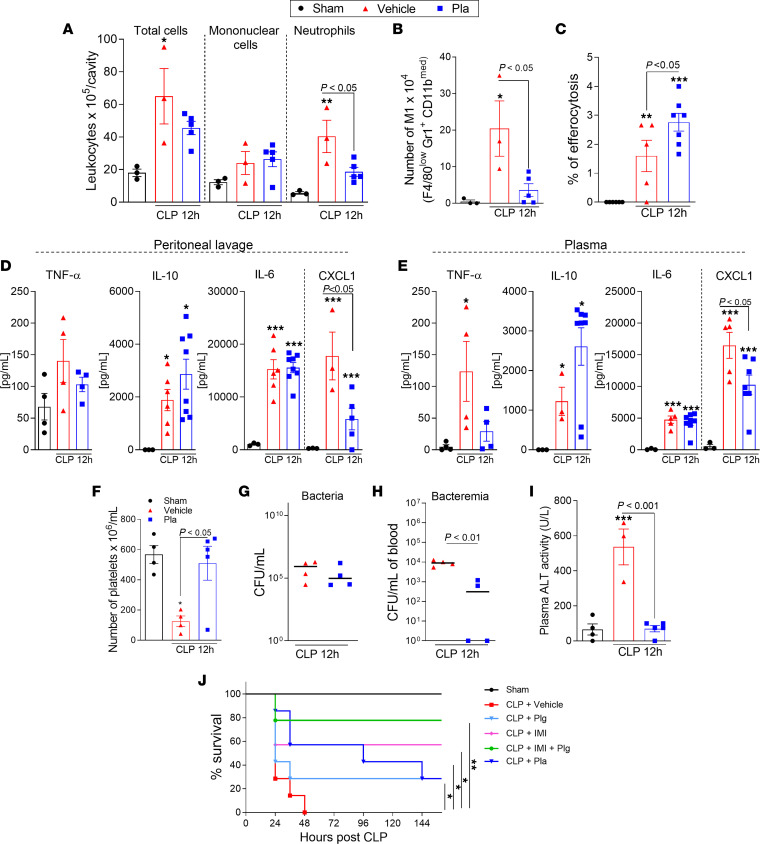
Effect of Pla treatment on inflammatory parameters and survival rates during severe sepsis induced by CLP. WT C57BL/6J mice (*n* = 4–8) were subjected to severe (18G needle) CLP and then treated with Pla (10 μg/mouse i.p.) 3 hours later. Cells present in the peritoneal cavity were harvested 12 hours after CLP. (**A** and **C**) The number of total cells, mononuclear cells, and neutrophils (**A**), and frequency of efferocytosis (**C**) were evaluated by counting cytospin slides treated with May–Grünwald–Giemsa stain. (**B**) The number of M1 (F4/80^low^ GR1^+^ CD11b^med^) macrophages were determined by flow cytometry. (**D** and **E**) The levels of TNF, IL-10, IL-6, and CXCL1 were quantified in cell-free peritoneal lavages (**D**) and plasma (**E**), respectively, by ELISA. (**F**) Platelets counted from blood samples. (**G** and **H**)The peritoneal fluid (**G**) and blood (**H**) samples were plated in brain–heart infusion medium for the analysis of bacterial load. (**I**) ALT activity was measured from plasma. Results are shown as the mean ± SEM or median of 4–8 mice per group. The experiments were performed 3 times with similar results. *P < 0.05, **P < 0.01, or ***P < 0.001 when comparing the sham group with the CLP group by 1-way ANOVA with post hoc Newman-Keuls (multiple groups) or unpaired 2-tailed Student’s *t* test (when comparing 2 groups). *P* values are indicated in the graphs when comparing vehicle with Pla-treated mice. Outliers were removed from the graphs when detected. In the survival experiments, C57BL/6J mice (*n* = 7) were subjected to severe (18G needle) CLP and treated with Pla (10 μg/mouse, i.p.), Plg (10 μg/mouse, i.p.), imipenem (IMI; 30 mg/kg, i.p.), or a combination of both (Plg 10 μg/mouse i.p. + IMI 30 mg/k, i.p.) after 3 and 12 hours of sepsis induction. (**J**) The survival rates were monitored for 6 days. The experiment was performed 2 times with similar results. *P < 0.05 when comparing vehicle-treated mice with Pla-, Plg-, or IMI-treated mice. **P < 0.01 when comparing vehicle-treated mice with Plg- + IMI-treated mice by log-rank test.

Next, we questioned whether the systemic administration of Pla might have therapeutic benefit in sepsis. Mice were subjected to severe sepsis and treated with Pla (10 μg/mouse, i.p.) 3 hours after the onset of inflammation (34). Inflammatory parameters and bacterial loads were evaluated 12 hours after CLP. Exogenous Pla administration significantly decreased the numbers of neutrophils (Figure 4A) and M1 macrophages (Figure 4B) in the peritoneal cavity, without changing total mononuclear cell numbers (Figure 4A) at the infectious site. Interestingly, Pla-induced reduction of neutrophil numbers was accompanied by increased efferocytosis of apoptotic neutrophils (Figure 4C) and decreased local (Figure 4D) and systemic (Figure 4E) levels of the neutrophil chemokine CXCL1. The levels of TNF, IL-10, and IL-6 in the peritoneal lavage (Figure 4D) and plasma (Figure 4E) were not altered by Pla treatment. In keeping with positive correlation between Plg levels and platelets (Supplemental Table 2), the treatment with Pla rescued the thrombocytopenia characteristic of sepsis (Figure 4F). No significant differences in bacterial counts were found in the peritoneal fluid (Figure 4G); however, reduced bacteria counts were observed in the blood of Pla-treated mice relative to vehicle-treated animals (Figure 4H). Notably, Pla treatment increased bacterial phagocytosis by macrophages in vitro (Supplemental Figure 4).

Next, we assessed whether Pla treatment of severely septic mice could afford protection against sepsis-induced organ damage. High levels of ALT were found in mice with severe sepsis and these were decreased after Pla treatment (Figure 4I). Because our data showed beneficial effects of Pla administration in severe sepsis, we tested the hypothesis that Pla, given as the active enzyme or as the zymogen Plg, would protect mice from sepsis lethality. For this purpose, Pla or Plg was given twice to mice, with an interval of 9 hours between injections. As seen in Figure 4J, administration of Pla or Plg could rescue approximately 30% of mice from lethal sepsis. Notably, Plg, in combination with imipenem, significantly enhanced mice survival when compared with the group that only received antibiotic treatment (80% versus 60% survival, respectively;) (Figure 4J). These results suggest that Pla reduced inflammation and protected mice from the lethality of severe sepsis.

Because Plg administration led to protective effects that were similar to those of active Pla, we next wondered whether the urokinase-type plasminogen activator (uPAR) would be important to limit the severity of sepsis. uPAR is an important regulator of the Plg system; it binds and activates the serine protease urokinase-type plasminogen activator (uPA; also known as urokinase). Activated uPA cleaves the zymogen Plg, generating the protease Pla (35). WT and uPAR^–/–^ mice were subjected to nonsevere sepsis. Interestingly, like Plg^–/–^ mice, uPAR^–/–^ mice exhibited increased lethality (51%) when compared with WT mice (100% survival); the lethality was associated with exacerbated inflammation (Supplemental Figure 3). The results in uPAR-deficient mice suggest that the conversion of Plg to Pla is also an important mechanism to mediate Pla protective effects in septic mice.

### Plg-deficient mice are more susceptible to LPS-induced endotoxemia, and Pla-treatment of endotoxemic WT mice decreases inflammatory parameters and fibrinogen levels.

**Figure 5:**
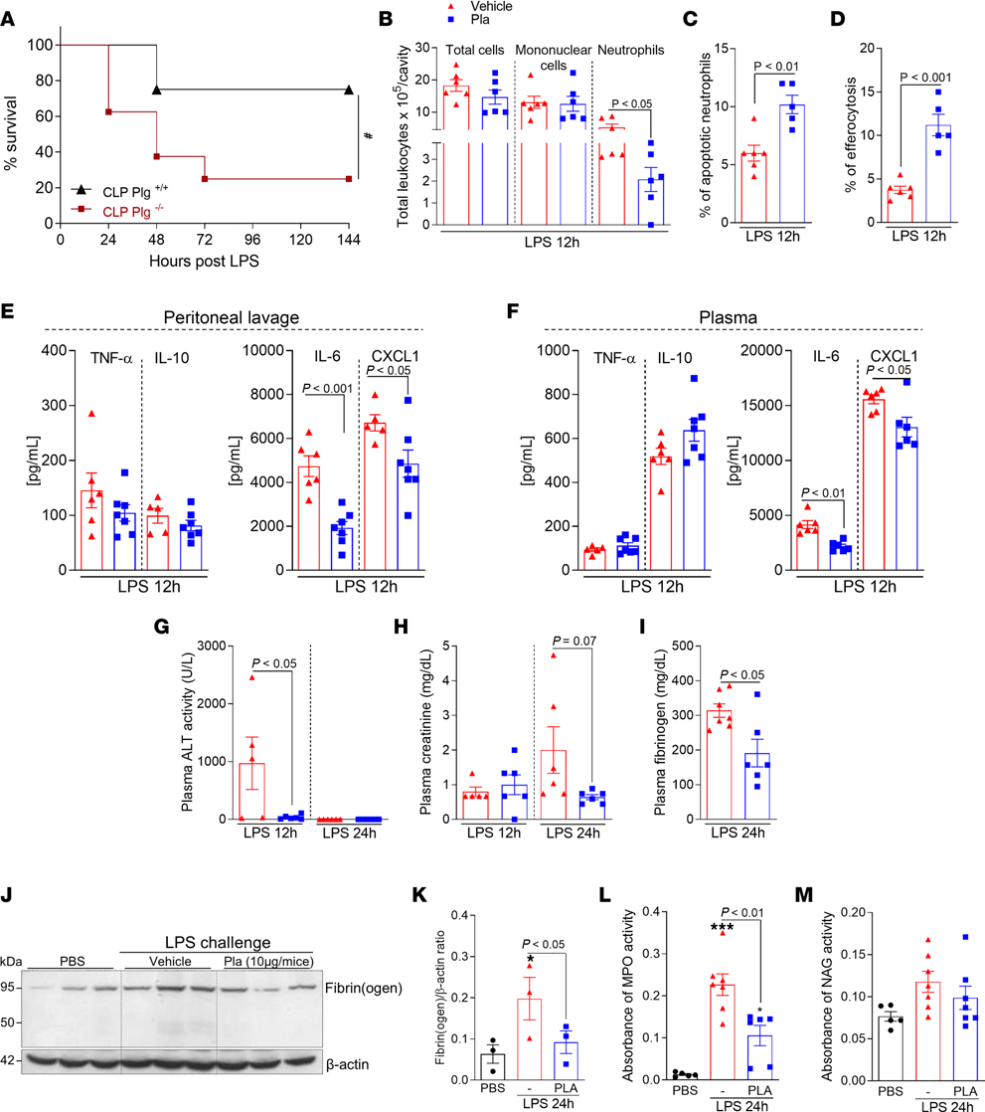
Effect of Plg depletion on lethality and of Pla treatment on inflammatory parameters during endotoxemia induced by LPS. Plg^+/+^ and Plg^–/–^ mice were subjected to an i.p. injection of LPS (10 mg/kg). (**A**) The survival rates (*n* = 8 mice) were monitored for 6 days. ^#^*P* < 0.05 when comparing Plg^+/+^ and Plg^–/–^ mice by log-rank test (survival curves). WT C57BL/6J mice were subjected to an i.p. injection of LPS (10 mg/kg) and then treated with Pla (10 μg/mouse i.p.) 3 hours later. Cells present in the peritoneal cavity were harvested 12 hours after LPS injection. (**B**–**D**) The number of total cells, mononuclear cells, and neutrophils (**B**), percentage of apoptotic neutrophils (**C**), and efferocytosis (**D**) were evaluated by counting cytospin slides treated with May–Grünwald–Giemsa stain. (**E** and **F**) The levels of TNF, IL-10, IL-6, and CXCL-1 were quantified in cell-free peritoneal lavages (**E**) and plasma (**F**), by ELISA. (**G–I**) The ALT activity (**G**), and creatinine (**H**) and fibrinogen (**I**) levels were measured in plasma. (**J**) Expression of fibrin(ogen) in the liver was determined by Western blotting (*n* = 3) with anti–β-actin used as loading control. (**K**) Densitometric analysis is also represented. (**L** and **M**) MPO (**L**) and NAG (**M**) activities were measured in the liver homogenates. Results are shown as the mean ± SEM of 5–7 mice per group. The experiments were performed 3 times with similar results. *P < 0.05, ***P < 0.001 when comparing PBS-injected mice with LPS-injected mice. *P* values are indicated in the graphs when comparing vehicle with Pla-treated mice by 1-way ANOVA with post hoc Newman-Keuls (multiple groups) or unpaired 2-tailed Student’s *t* test (when comparing 2 groups). Outliers were removed from the graphs when detected.

We further examined the protective role of Plg/Pla in a model of endotoxemia induced by LPS. First, Plg^+/+^ and Plg^–/–^ mice were injected with LPS (10 mg/kg, i.p) to induce sepsis, and the survival rates were evaluated for 6 days (Figure 5A). As in the CLP model, Plg^–/–^ mice exhibited increased lethality (75%) when compared with their Plg^+/+^ littermates (25%).

Next, the effect of Pla treatment was evaluated in WT mice. Similar to our results in the severe CLP model, Pla treatment of mice with endotoxemia reduced the numbers of neutrophils without significant changes in total leukocyte and mononuclear cell numbers (Figure 5B). Interestingly, Pla treatment promoted neutrophil apoptosis and macrophage efferocytosis (Figure 5, C and D). In keeping with the Pla-induced reduction of neutrophil numbers, the levels of CXCL1 were decreased in both peritoneal lavage (Figure 5E) and plasma (Figure 5F). Interestingly, Pla treatment decreased local and systemic levels of IL-6 but not of TNF and IL-10 (Figure 5, E and F). In addition, markers of liver and kidney damage or dysfunction were assessed in plasma at 12 and 24 hours after LPS administration. ALT levels were significantly reduced after Pla treatment at 12 hours (Figure 5G), and there was a trend toward reduction in creatinine levels at 24 hours (Figure 5H). We also examined the plasma levels of fibrinogen and found their levels were decreased after Pla treatment (Figure 5I).

Moreover, fibrin(ogen) deposition in the liver was also reduced after Pla treatment (Figure 5, J and K). Leukocyte accumulation in the liver was evaluated by myeloperoxidase (MPO) and *N*-acetylglucosaminidase (NAG) activities (indirect measurements of neutrophils and macrophages, respectively). Pla treatment reduced MPO activity (Figure 5L), but no changes were observed in NAG (Figure 5M). Altogether, these results reinforce the protective role of Pla during systemic inflammatory conditions by decreasing the overall inflammatory response and fibrinogen levels or deposition and reducing organ damage.

### Plg and Pla prevent NETs release.

Neutrophil extracellular traps (NETs) are structures composed of granules containing cytotoxic enzymes, including MPO and elastase, and nuclear constituents (DNA matrices containing histone) that are released extracellularly, ensuring a high local concentration of antimicrobial agents to degrade virulence factors and kill bacteria (36). However, during sepsis, there is an excessive release of NETs, which contribute to the development of organ failure and have been correlated with sepsis severity (37). To further explore the mechanism by which Plg and Pla protect mice from sepsis severity, in vitro and in vivo evaluation of NETs was performed.

**Figure 6:**
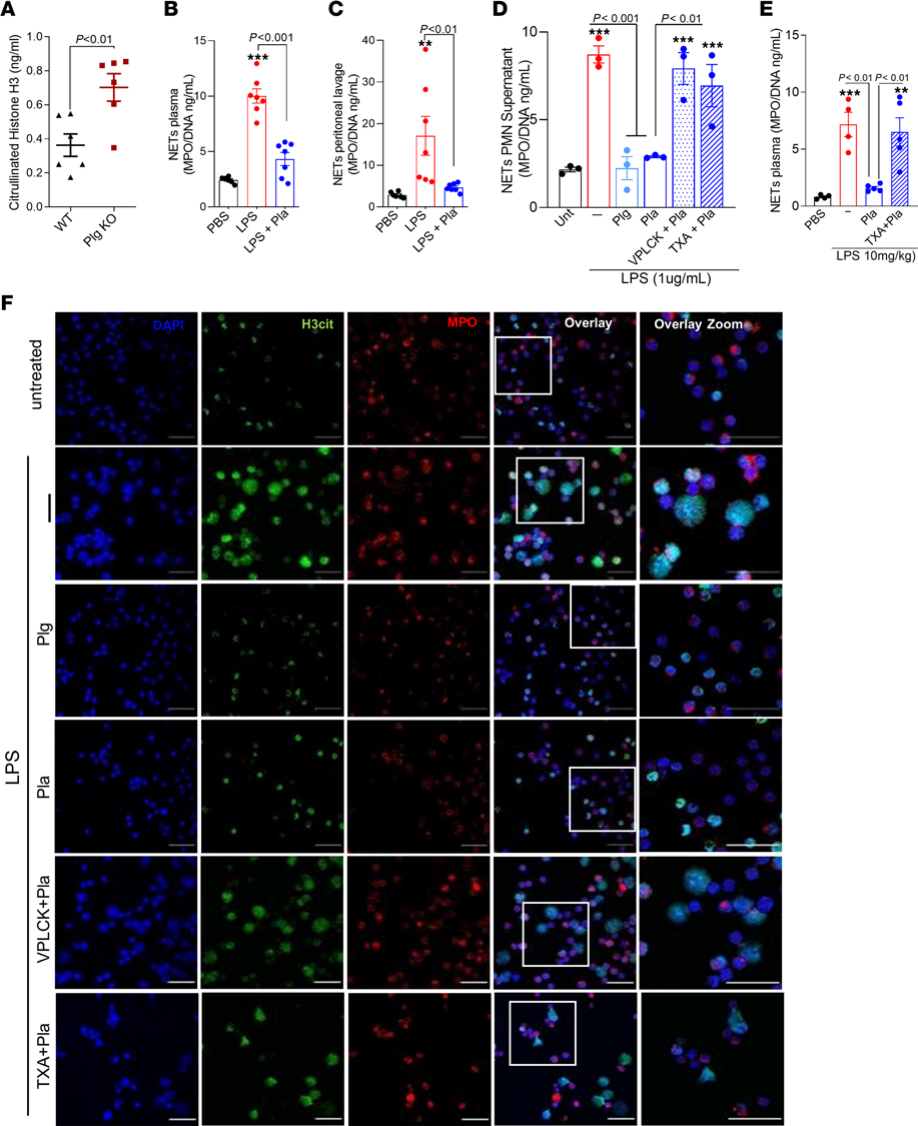
Effects of Plg and Pla on NETs release in vivo and in vitro. (**A**) Plg^+/+^ and Plg^–/–^ mice (*n* = 6) were subjected to nonsevere (30G needle) CLP and the plasma levels of H3cit were determined by ELISA. WT C57BL/6J mice (*n* = 4–7) were subjected to an i.p. injection of LPS (10 mg/kg) and then treated with Pla (10 μg/mouse i.p.) 3 hours later. (**B** and **C**) NETs release (MPO/DNA) in plasma (**B**) and peritoneal lavages (**C**) was determined 12 hours after LPS injection. Bone marrow neutrophils obtained from C57BL/6J mice were pretreated with Plg (4 μg/mL), Pla (4 μg/mL), or with Pla preincubated with the inhibitors (D-Val-Phe-Lys chloromethyl ketone [VPLCK] 22.5 nM or TXA 0.1 M) by 1 hour before stimulation with ultrapure LPS (1 μg/mL) for an additional 4 hours. (**D**) Quantification of NETs release (MPO/DNA) in supernatant. (**E**) WT C57BL/6J mice were subjected to an i.p. injection of LPS (10 mg/kg) and then treated with Pla (10 μg/mouse, i.p.) 3 hours later. TXA (100 mg/kg, i.p.) was injected 30 minutes before Pla. Plasma was collected 12 hours after LPS injection for NETs release (MPO/DNA) measurement by ELISA. **P < 0.01, ***P < 0.001 when comparing untreated (Unt) or PBS-injected mice with LPS-stimulated or injected and treated groups. *P* values are indicated in the graphs when comparing CLP-WT versus CLP-KO mice (unpaired 2-tailed Student’s *t* test) or vehicle with Pla-treated mice/cells by 1-way ANOVA with post hoc Newman-Keuls (multiple groups). Outliers were removed from the graphs when detected. (**F**) Representative fluorescence images of NETs stained for DNA (DAPI, blue), H3cit (green), and MPO (red) are shown. Scale bar: 50 μm at ×630 magnification.

The levels of citrullinated histone H3 (H3cit), a marker of NET extrusion, were higher in plasma of septic Plg^–/–^ mice compared with that of Plg^+/+^ littermates (Figure 6A). In addition, Pla treatment of WT endotoxemic mice resulted in reduced levels of NETs (as determined by MPO/DNA conjugates) in plasma (Figure 6B) and in the peritoneal lavage (Figure 6C).

Because Plg deficiency was associated with higher levels of NETs and Pla treatment of WT mice reduced NETs during sepsis, we tested the effect of Plg and Pla directly on NETs formation in mouse neutrophils in vitro to avoid bias related to increased numbers of neutrophils found in septic Plg^–/–^mice. Interestingly, although LPS induced significant release of NETs by neutrophils, pretreatment of cells with Plg or Pla prevented extrusion of NETs after LPS stimulation (Figure 6, D and F). To investigate whether the inhibition of NETs formation by Pla was dependent on its proteolytic activity and binding to lysine sites exposed in Plg/Pla receptors, we tested the effect of a selective Pla inhibitor, D-Val-Phe-Lys chloromethyl ketone, and the lysine analog tranexamic acid (TXA). These experiments with inhibitors were carried out with Pla because Plg, the zymogen form of Pla, is not endowed with protease activity. Pla inhibition prevented NETs release in vitro (Figure 6, D and F) and in vivo (Figure 6E), denoting a requirement for the proteolytic activity of Pla and its binding to cell surfaces for Pla inhibition of NETs formation. Interestingly, our data showed that the decrease in NET release can be achieved after Plg pretreatment (Figure 6D), because Plg is known to bind to the cell surface and it is further converted to Pla. As a control, treatment with Pla or Plg alone did not lead to NET extrusion by murine neutrophils (Supplemental Figure 5).

Altogether, these data suggest that Plg/Pla afforded significant protection from sepsis lethality, with promising therapeutic value to be further investigated in clinical studies.

## Discussion

**Figure 7:**
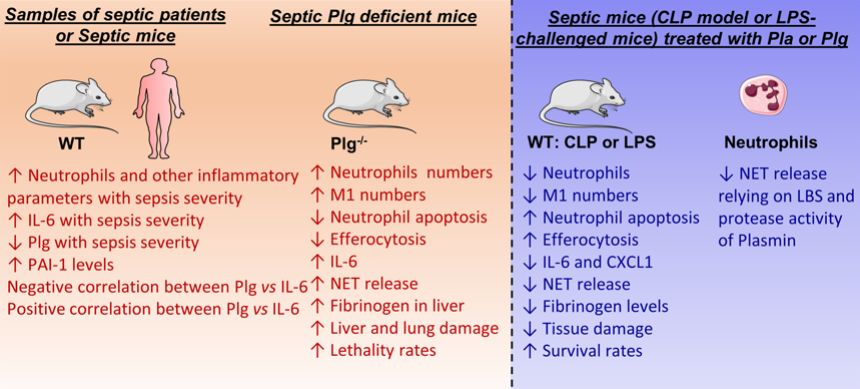
Summary of the main findings of Plg/Pla in sepsis from our in vivo and in vitro studies. LBS, lysine binding sites.

Sepsis is a systemic inflammatory syndrome triggered by a given infection that can lead to multiple-organ dysfunction and disseminated intravascular coagulation (38). In addition to the classical role of Plg/Pla in the dissolution of fibrin clots in vivo (39), the fibrinolytic system has also been associated with a diverse array of biological activities, including the regulation of cell migration, tissue repair, and inflammation (20, 28). Indeed, accumulating evidence has demonstrated a role for Plg/Pla in modulation of inflammation in vitro (24, 26, 27) and in preclinical models of self-resolving inflammation (21–23). Here, we provide evidence suggesting that Plg/Pla could be a beneficial adjuvant for the treatment of sepsis by improving the overall inflammatory unbalanced response, decreasing NET release, fibrinogen levels or deposition, preventing tissue damage, and reducing mortality, all of which are summarized in Figure 7.

The cytokine storm is a key feature of sepsis (40). During tissue injury caused by an infectious agent, inflammatory mediators, including cytokines, are released, which, in turn, can induce additional tissue damage and production and release of additional cytokines, in a positive-feedback manner (41). Among several soluble cytokines, IL-6 has been reported to be a major prognostic marker of sepsis severity (42, 43). Here, our experimental model of sepsis recapitulated this key feature of sepsis (e.g., Figure 7). IL-6 levels were significantly higher in mice subjected to severe sepsis compared with the group with nonsevere sepsis. Notably, deficiency of Plg led to an overall worse sepsis outcome, which was accompanied by elevated levels of IL-6 when compared with Plg-sufficient mice. Moreover, Plg levels were significantly reduced in patients with sepsis shock when compared with levels in patients with sepsis, supporting the concept that severity of sepsis is related to reduced Plg levels. Importantly, our results showed that plasma levels of IL-6 were negatively correlated with Plg levels in both humans and mice. Furthermore, harnessing the Plg/Pla pathway through systemic treatment with either Plg or Pla was protective in 2 different models of sepsis (infectious and LPS-induced). Of importance was the reduction of IL-6 levels by Pla treatment during LPS-induced endotoxemia, but IL-6 levels were not altered when Pla was given as treatment to mice subjected to CLP. These differences may relate to the overall higher inflammatory response triggered by an active polymicrobial infection (e.g., CLP-induced sepsis) compared with LPS-induced endotoxemia.

Upon a given injury or infection, coagulation events are rapidly triggered to prevent hemorrhage and contain microorganisms. The activation of the coagulation cascade also triggers fibrinolysis to avoid excessive coagulation; therefore, initially increased levels of Plg and Pla are observed during injury (32). On the other hand, uncontrolled systemic infection or inflammation, as observed in sepsis, leads to the consumption of fibrinolysis components over time and triggers the sustained and increased production of fibrinolysis inhibitors (18). Indeed, sepsis is associated with coagulation abnormalities such as disseminated intravascular coagulation and a significant reduction in fibrinolysis. This effect is due, in part, to increased levels of PAI-1, an important inhibitor of fibrinolysis (18). In our human cohort of patients with septic shock, we found lower Plg levels and substantial amounts of PAI-1, reinforcing the notion of a sustained impairment of fibrinolysis on sepsis severity (17). Another mechanism associated with the fibrinolysis defects in sepsis is the degradation of Plg mediated by neutrophil elastase, especially in patients with sepsis shock (44). In addition, an important consequence of sepsis is the systemic inflammation, intense tissue damage, and, consequently, organ failure. In this sense, the liver is strongly affected, compromising its biological functions, including the production of Plg. Here, a combination of these events may contribute to the reduction of Plg levels observed, especially in severe sepsis.

Recently, it was demonstrated that granulocyte microvesicles with a high Pla-generation capacity promote clot lysis and improve outcome in septic shock (45). Mechanistically, this process involves the binding and activation of Plg to Pla by uPA/uPAR present at the microvesicle surface. It was also reported by the same research group that patients with septic shock with increased levels of microvesicles with a high Pla-generation capacity have a higher survival rate compared with patients with a lower level (46). Here, Plg^–/–^ and uPAR^–/–^ mice had reduced survival rates after CLP. Interestingly, Plg levels increased over time during the recovery of hospitalized patients with sepsis. In contrast, patients with more severe disease (septic shock) did not present the same increase over time and, from the day of admission, had lower levels of Plg and high activity of PAI-1 in comparison with patients who had less severe sepsis. Our data are consistent with those obtained from a cohort of patients with COVID-19 who were aged and/or had high-risk comorbidities, in whom low levels of Plg were associated with worse prognostic parameters such as higher levels of IL-6, CRP, and markers of organ dysfunction (47). Therefore, the human data reinforce the clinical relevance of our findings in the CLP-induced sepsis and LPS-induced endotoxemia models.

The early stage of sepsis is characterized by an exacerbated inflammatory phase known as systemic inflammatory response syndrome, during which there is a massive release of several proinflammatory mediators and intense neutrophil recruitment to the affected tissue, causing severe tissue damage (48). Here, we show that mice subjected to sepsis (via CLP or endotoxemia) and treated with Pla exhibited a significant reduction in the number of neutrophils recruited to the peritoneal cavity and increased efferocytosis. In keeping with that, we previously showed, in an acute model of pleurisy induced by LPS, that pharmacological treatment of inflamed mice with Pla reduces neutrophil accumulation in the pleural cavity (21). This was associated with the capacity of Pla to induce neutrophil apoptosis and promote efferocytosis (21). Indeed, several studies have reported a pivotal role of Pla in the clearance of apoptotic cells (21, 22, 24–26). This effect may explain, in part, the reduction in the numbers of neutrophils accumulated in the peritoneal cavities of Pla-treated mice observed in the present study.

Although other studies have shown that Plg/Pla does not affect neutrophil recruitment in simple models of self-resolving inflammation (21–23, 49), such as pleurisy and peritonitis elicited by thioglycolate, neutrophil recruitment is affected in more complex models of severe inflammation (50, 51), as in the preclinical models of sepsis used in our study, in which neutrophil accumulation was exacerbated in Plg^–/–^ and uPAR^–/–^ mice after CLP. To exploit the therapeutical potential of Pla, we have shown, in septic mice, that Pla administration reduced neutrophil numbers associated with decreased levels of the neutrophil-chemoattractant chemokine CXCL1 and increased neutrophil apoptosis and their removal by efferocytosis. Consistent with our findings of increased neutrophil recruitment and activation in septic Plg^–/–^ mice, in a model of infection by *Mycobacterium avium*, Plg^–/–^ mice had earlier bacterial dissemination to organs, with enhanced fibrin and fibronectin deposition, associated with increased neutrophil infiltration within liver granulomas (51). Indeed, a recent report has highlighted the critical role of microbiota-induced fibrin deposition at the oral mucosa, with consequent neutrophil activation, which becomes tissue damaging in the settings of Plg deficiency in mice and humans (50).

An important feature of the resolution of inflammation is the shift in macrophage phenotype from M1-like (pro-inflammatory) to M2-like (anti-inflammatory) (52, 53). M2-like macrophages clear apoptotic neutrophilic infiltrates at inflammatory sites, preventing secondary necrosis and exacerbation of inflammation (54). Regulatory macrophage recruitment and polarization can be induced by different proresolving mediators (55), which enhance the ability of macrophages to phagocytose bacteria, reducing inflammation and improving survival of mice in infectious models, including CLP-induced sepsis (56, 57). Plg^–/–^ mice display increased frequency of the pro-inflammatory M1 macrophages during acute inflammation, consistent with the capacity of Plg and Pla to skew macrophages toward proresolving phenotypes (21, 22). Here, we found increased numbers of M1 macrophages in Plg^–/–^ and uPAR^–/–^ mice subjected to CLP. Most importantly, Pla treatment of septic mice decreased the numbers of these pro-inflammatory macrophages. Given the detrimental role of accumulation of M1 macrophages in sepsis (58) and that reduction of their numbers or shifting toward proresolving phenotypes is an underlying mechanism of protection afforded by proresolving mediators in sepsis (56, 57), we suggest that this reduction of M1 numbers is a protective factor that could contribute to the overall protection of Pla-treated mice.

To our knowledge, our study is the first to investigate the role of Plg in polymicrobial sepsis (induced by CLP). The CLP has been considered the gold standard sepsis model and has been widely used over the past 40 years. It is the most frequently used model because it closely resembles the progression and characteristics of human sepsis (59). In the CLP model, animals display many characteristics that are also seen in humans, such as overwhelming systemic inflammation that causes tissue damage and further organ dysfunction. In addition, mice also display disease patterns with typical signs of septic shock, such as hypothermia, hypotension, tachycardia, and tachypnea (60). In our experimental settings, the signs of disease were worse in Plg^–/–^ mice, including inflammatory response with higher NET release, fibrin(ogen) deposition in the liver, organ damage, and increased lethality rates. Moreover, Plg^–/–^ mice had signs of loss of vascular integrity, as seen by a trend for hemorrhage and edema observed in the lungs. In a previous study of monomicrobial sepsis using a bacterial infection model induced by *Staphylococcus aureus*, although survival in Plg^–/–^ mice was reduced after inoculation of 1×10^7^ CFU of *S*. *aureus* (61), these mice had increased survival rates when given a higher concentration of inoculum (1.6×10^8^ CFU). Differences in the biology of bacteria in the infection model and the use of models of infection with single versus polymicrobial bacteria might be a potential explanation for the contrasting data. For instance, *S*. *aureus* is known to manipulate fibrinolysis to promote bacterial invasion and spread (62). Conversely, results of a monomicrobial (10^4^ CFU of *Escherichia coli*) i.p. infection showed that Plg deficiency did not alter the host inflammatory and antimicrobial responses (63). Of interest, Pla increased phagocytosis of bacteria in cultured macrophages (Supplemental Figure 4).

An important neutrophil effector mechanism is the release of NETs, which are constituted of extracellular DNA, granule proteases, and histones, and are an important immune defense against microorganism proliferation and dissemination (36, 64). Although NET release by neutrophils is an important mechanism for host bacterial control, sustained release of NETs has been described to contribute to the pathogenesis of many diseases, including sepsis (37, 65–67). In addition, augmented NET levels are correlated with sepsis severity (9, 37, 68) and with COVID-19 pathology (67), and can be pharmacologically degraded or removed by rhDNase I treatment. Interestingly, a recent study showed that increased neutrophil accumulation and NET formation mediated periodontal immunopathology in Plg^–/–^ mice (50). In that elegant study, the impaired fibrinolysis in Plg^–/–^ mice and consequent fibrin accumulation led to persistent activation of neutrophils and NETs formation, an effect that was reduced with inhibition of NETs by systemic DNase I treatment. Noteworthy, no changes in the oral microbiota structure and composition were observed in Plg^–/–^ mice when compared with their littermates (50). Here, we show that septic Plg^–/–^ mice had higher numbers of neutrophils and defective neutrophil apoptosis associated with high levels of Hcit3 in plasma and increased fibrin(ogen) in the liver, consistent with augmented systemic release of NETs. Importantly, Plg and Pla prevented NET release in endotoxemic mice and in murine neutrophils stimulated with LPS. Pla modulation of NETs in vitro required its protease activity and ligation to cellular receptors (mediated by lysine binding sites). In addition, neutrophil apoptosis induced by Plg/Pla (21) may be the underlying mechanism by which Plg/Pla reduces NET release, like that reported for the proresolving Annexin A1 peptide (9). Akin to these findings, reduced neutrophil apoptosis was observed in Plg^–/–^ septic mice. Therefore, here we uncovered mechanisms for the protective effects of Pla/Plg in sepsis. Overall, our data demonstrate a key role of the Plg/Pla system in infectious disease and suggest Plg/Pla administration as a promising adjunctive therapy for treating sepsis.

## Methods

### Mice.

Male C57BL/6J mice (6–8 weeks old) were obtained from Centro de Bioterismo, Universidade Federal de Minas Gerais (UFMG), and the Animal Facility of the Scripps Research Institute. Plg^–/–^ were a gift from Victoria Ploplis (University of Notre Dame, IN, USA). Plg^–/–^ and Plg^+/+^ littermate controls were 6–7 weeks old and were kept in cohousing. Mouse genotypes from tail biopsy specimens were determined using real-time PCR with specific probes designed for each gene (Transnetyx, Cordova, TN). The mice are in the C57BL/6J background, and heterozygotes were mated to obtain WT and KO littermates. uPAR-deficient mice were in the C57BL/6J background (69). Mice were maintained under controlled temperature and lighting with free access to filtered water and food. Mice were randomly assigned to groups and the experiments were carried out in a blinded fashion. All procedures described here had prior approval of the IACUC of the Scripps Research Institute and the Ethics Committee in Animal Experimentation of the UFMG.

### Drugs, reagents, and Abs.

Detailed information is provided in Supplemental Methods.

### Polymicrobial sepsis model and endotoxemia model.

The induction of sepsis was performed by CLP and adapted from previous studies (70, 71). Mice were anesthetized by i.p. administration of ketamine (80 mg/kg) and xylazine (10 mg/kg). Under aseptic conditions, the cecum was externalized and ligated below the ileocecal valve. A double puncture was made through the cecum with an 18G or 30G needle to induce severe or nonsevere sepsis, respectively. The cecum was gently squeezed to expel a small amount of feces and returned to the peritoneal cavity. The abdominal incision was sutured and immediately after surgery, 1 mL of saline was administered s.c. for fluid resuscitation. Sham-operated mice were treated as above without ligation or puncture of the cecum. For the endotoxemia model, mice were injected with LPS (10 mg/kg, i.p). Treatments are described in Supplemental Methods. Human Plg and Pla were from Calbiochem (EMD Chemicals) and Sigma-Aldrich, respectively.

### Leukocyte recruitment to the peritoneal cavity.

Leukocyte recruitment was assessed 12 hours after either initiating the CLP model or administering LPS injection (10 mg/kg, i.p.). Mice were euthanized and the peritoneal cavity was irrigated with 4 mL of sterile PBS/EDTA (1 mM). Total leukocyte counts were performed in Neubauer chambers with an optical microscope. Differential cell counts (neutrophils and mononuclear cells) were performed on cytospin slides from peritoneal fluid stained by the May–Grünwald–Giemsa method.

### Apoptosis and efferocytosis.

Apoptosis and efferocytosis were determined as described previously (21, 22) and in Supplemental Methods.

### Phagocytosis and bacterial counts.

Phagocytosis and bacterial counts were determined as described previously (72) and as described briefly in Supplemental Methods.

### NET quantification.

MPO/DNA assays and measurement of H3cit were performed as previously described (37, 68, 73) and as described in Supplemental Methods.

### Immunofluorescence staining and confocal microscopy.

Immunofluorescence staining and confocal microscopy were performed in murine neutrophils as described (68) and as briefly described in Supplemental Methods.

### Patient samples.

Patients were participants in a prospective cohort of patients with sepsis who were admitted to the intensive care unit of the Hospital das Clínicas da UFMG. The study was approved by the Ethics Committee of the UFMG. Detailed procedures of patients’ clinical data and analyses in human samples are found in Supplemental Methods.

### ELISA and other kits.

Kits for cytokines, Plg, and PAI-1 or measurements of fibrinogen, ALT, and creatinine are listed in Supplemental Methods.

### Flow cytometry.

Cells collected from peritoneal fluid were analyzed by flow cytometry. M1 macrophages in peritoneal cavity were defined by the F4/80^low^ Gr1^+^ Cd11b^med^ population (21, 74).

### Statistics.

The description of the statistical analysis of mouse and human data is provided in the Supplemental Materials.

### Study approval.

Experiments had prior approval from the Animal Ethics Committee of UFMG (protocol no. 3/2019), the IACUC of the Scripps Research Institute (protocol no. 09-0042), and the Research Ethics Committee of UFMG for human studies (protocol no. 53351416.9.0000.5149). For analyses of human data, the participants provided written informed consent prior to participation in the study.

## Author contributions

JPV, RJP, LAM, and LPS designed the research and analyzed data. JPV, LPS, LPT, and LAM wrote the paper. JPV, IZ, LFB, LCRT, LOP, CMSS, TCM, EMM, ASB, MAS, LCG, AHS, and NB performed experiments. CMQJ performed histological analysis. INB and VN collected and provided patient samples. AT, RGF, JCAF, VN, MMT, and RJP provided essential tools and expertise.

## Supplementary Material

Supplemental data

## Version 1 03/14/2023

In-Press Preview

## Version 2 04/24/2023

Electronic publication
