# Supplementary material for: Plasmin and plasminogen prevent sepsis severity by reducing neutrophil extracellular traps and systemic inflammation
Source: JCI Insight. 2023 Apr 24;8(8):e166044. doi: 10.1172/jci.insight.166044 (PMC10243804; doi:10.1172/jci.insight.166044)
Supplement: Supplemental data [file jciinsight-8-166044-s184.pdf]

Materials and Methods

Drugs, reagents and antibodies

Human Plg and VPLCK (D-Val-Phe-Lys chloromethyl ketone) were from Calbiochem (EMD Chemicals, San Diego, CA, USA) human Pla and TXA (tranexamic acid) were from Sigma-Aldrich (St. Louis, MO, USA). LPS from *Escherichia coli* (serotype O:111:B4) was from Sigma-Aldrich. Fluorescent monoclonal anti-mouse antibodies for flow cytometry were F4/80 (PE-Cy7, Invitrogen, Carlsbad, CA, USA), GR1 (APC, eBioscience, Thermo Fisher Scientific, MA, USA), CD11b (V500, BD Biosciences, Franklin Lakes, NJ, USA).

Treatments

Mice received an intraperitoneal (i.p.) injection of either 10 µg of human plasmin, 10 µg of human plasminogen or vehicle. To analyse inflammatory parameters, mice received a single treatment 3 hours after sepsis induction. To analyse survival rates, mice were treated at 3 and 12 hours after sepsis induction. A group of mice also received a broad-spectrum antibiotic, imipenem (30 mg/kg i.p.) alone, or in combination with plasminogen (10 µg/mice). The doses of plasminogen or plasmin used here (10 µg/mice or 100 nM) were chosen after titration in our CLP model and are very similar to that we have used in our previous studies (2-4ug/cavity or 2-4 ug/ml or 20-40nM) (1-3) and from other studies (10-120nM) (4, 5). TXA (100mg/kg, i.p.) (6) was given 30 min prior to Pla.

Bacterial counts

Measurement of bacteria in blood and peritoneal lavage fluid was performed 12 hours after the induction of sepsis. Mice were euthanized and blood was collected by cardiac puncture using sodium citrate (3.2%). Peritoneal lavage was performed with 4mL of sterile PBS/EDTA (1mM). Bacterial loads were determined by plating blood and the

peritoneal lavage in Brain Heart Infusion (BHI) plates (37°C incubation, overnight). The results were expressed as log of CFU per mL.

Phagocytosis

Phagocytosis was evaluated as previously (7). Briefly, $2x10^5$ murine bone marrow-derived macrophages (BMDMs) isolated from WT C57BL/6J mice, were plated, and incubated with *E. coli* (MOI 1:10) for 3 hours to allow phagocytosis (1 h of adhesion at 4°C followed by 2 h at 37°C). Noninternalized bacteria were washed out with gentamycin (5 µg/ml in PBS, 30min.). To assess phagocytosed bacteria, macrophages were lysed as described (7, 8), and viable internalized bacteria were counted in LB agar plates after incubation (37°C, overnight).

Neutrophil preparation

Mouse bone marrow neutrophils were isolated using Percoll density gradients (1119 and 1077) and resuspended in RPMI 1640 supplemented with 0.5% BSA. Neutrophil purity was determined by cytospin slides (>95%).

NET quantification

Myeloperoxidase (MPO)/DNA assay was performed as previously described (9-11). Briefly, an antibody bound to a 96-well plate captured anti-myeloperoxidase (MPO - 5 µg/ml; Abcam). The amount of DNA bound to the enzyme was quantified using the Quant-iT™PicoGreen® kit (Invitrogen, MA, USA) according to the manufacturer's instructions. The fluorescence intensity (excitation at 488 nm and emission at 525 nm) was quantified using a FlexStation 3 Microplate Reader (Molecular Devices, CA, USA). Measurement of citrullinated histone H3 (H3cit) was performed according to the manufacturer's instructions (Cayman Chemical, MI, USA).

Immunofluorescence staining and confocal microscopy

The procedure was performed as described (11). Initially, neutrophils were attached to slides coated with a poly-L-lysine (0.1%) solution (Sigma-Aldrich, MO, USA) for 4 h and then fixed with paraformaldehyde (2%). The samples were then washed with wash buffer and blocked with 1% BSA and glycine (22.52 mg/mL) in PBST (PBS + 0.1% Tween 20). The cells were stained with the following antibodies: rabbit anti-histone H3 (H3Cit; #ab5103 - Abcam; 1:500); mouse anti-MPO; 2C7; ab25989 - Abcam; 1:500). Next, the samples were incubated with the secondary antibodies anti-mouse IgG Alexa Fluor 647 (Abcam; 1:800); anti-rabbit IgG Alexa Fluor 594 (Abcam; 1:800). The nuclei were stained with DAPI; D1306 - Life Technologies; 1:1.000). Images were acquired using Axio combined with an LSM 780 confocal microscope system at 630× magnification (Carl Zeiss, Germany). All acquired images were analyzed using Fiji by ImageJ.

Patient samples

Peripheral blood samples from 12 septic and 10 septic shock patients were obtained in this study. The definitions proposed by the Third International Consensus Definitions for Sepsis and Septic Shock were applied (12). Patients admitted with confirmed or suspected severe sepsis or septic shock diagnosed at the time of the admission in the ICU or immediately after the diagnosis of sepsis, in case they were already at the ICU, were considered for inclusion. The inclusion criteria were: 1) age over or equal to 18 years old; 2) patients being hospitalized in one of the units participating in the study, with a perspective of staying hospitalized longer than 24 hours; 3) authorization to participate in the study through the signature of the informed consent (which could be done by a responsible person, if the patient was not aware or with adequate conditions of discernment); 4) patient's with clinical suspicion or microbiological confirmation of

sepsis or septic shock (12). Sepsis was defined by the presence of at least one new organ dysfunction associated with infection, characterized by a variation of at least two points in the Sepsis Organ Failure Assessment (SOFA) score (13). Septic shock was defined as persistent hypotension requiring vasopressors to maintain a mean arterial pressure (MAP) greater than 65 mmHg associated with a lactate serum level greater than 2 mmol/L despite adequate fluid resuscitation (14). Microbiological confirmation of the infectious condition was defined by the isolation of at least one microorganism considered pathogenic, no more than 48 hours before inclusion in the study, and for which physicians directed antimicrobial therapy. The clinical data were collected prospectively, and the following variables were recorded: age; sex; SOFA severity score at admission; total leukocytes, neutrophils and platelets counts; arterial lactate; C-reactive protein (CRP) levels; and hemoglobin levels.

The exclusion criteria were: 1) use of antibiotic therapy against the current infectious process for longer than 48 hours; 2) severely immunosuppressed patients (HIV infection with CD4+ lymphocyte levels < 200/mm^3, severe neutropenia < 500/mL, post-solid organ or bone marrow transplant patients, patients using chemotherapeutic agents in the last 28 days, patients with inflammatory or autoimmune diseases in chronic use of immunosuppressants or in pulse therapy; 3) patients undergoing palliative care only; 4) patients expected to be deceased in the next 24 hours; 5) Patients diagnosed with infections known to require prolonged antibiotic therapy; 6) polytraumatized patients or patients submitted to major surgeries in the last five days (except surgery for control of the infectious focus).

Blood samples were obtained at time of inclusion (day 1) and at the third day of follow-up. The serum samples were obtained from blood collected for routine ICU analyses. The serum was stored in a -80ºC freezer until analysis.

ELISA

The levels of murine IL-6, TNF-α, IL-10 and CXCL-1 were measured in the supernatants of peritoneal lavage fluids and plasma of mice by ELISA, according to the manufacturer's instructions (R&D Systems, MN, EUA). Human IL-6 measurement was performed in plasma of samples using Peprotech (New Jersey, USA) ELISA kit, according to the manufacturers' instructions. Plg measurement was performed on plasma of mice and from human cohorts by using ELISA kits from Abcam (UK). PAI activity assay was determined in serum of patients by ELISA (Molecular Innovations, MI, USA), according to the manufacturer's instructions.

ALT, creatinine and fibrinogen measurements

ALT (Gold Analisa Diagnóstica, MG, Brazil), fibrinogen (Bioclin, MG, Brazil), and creatinine (Labtest, MG, Brazil) levels were determined in plasma of mice according to the manufacturer's instructions. The results were expressed as indicated in graphs.

Western blotting

Liver tissue from mice were processed and lysed in Tissue Extraction Reagent (Invitrogen #FNN0071) containing Protease Inhibitor cocktail (Thermo Fisher Scientific). Lysates were electrophoresed on Bolt 4-12% Bis-Tris Plus polyacrylamide-SDS gels under reducing conditions and electrotransferred to nitrocellulose membranes. Membranes were incubated with primary antibodies (anti-Fibrinogen - Abcam ab92572; anti-β-actin - LI-COR, Lincoln, NE, USA), washed with PBS-Tween-20 0.1% and incubated with species specific IRDye®680RD/800CW or peroxidase-conjugate antibodies. Immunoreactive bands were visualized using the Odyssey Imaging System (LI-COR), or ECL detection system (GE Healthcare). For densitometric analysis, membranes were scanned and quantified using the software Image Studio™ Lite Software 5.2 (LI-COR).

MPO and NAG quantification

Livers were processed with different buffers specific for measurement of myeloperoxidase (MPO) or N-acetyl-β-D-glucosaminidase (NAG) activities used as neutrophil and macrophage accumulation indexes respectively, as described previously (8, 15).

Statistical analysis

All statistical analyses were performed using Prism 6.0 software. The results were presented as mean ± Standard Error of the Mean (SEM) per group of 3 to 8 animals. Results were analysed using the parametric t-test or non-parametric Mann Whitney test (two groups) or one-way analysis of variance (one-way ANOVA), followed by multiple comparisons of Newman-Keuls (three or more groups). Survival curves were expressed as percentage of live mice observed at 12-hour intervals for six days. The logrank test was used for the statistical analysis of the survival curves. $P < 0.05$ was considered as statistically significant. Outliers were excluded from the analyses.

Patient data analysis was performed using the SPSS Statistics software version 17.0 (SPSS Inc., Chicago, Ill., USA). We assumed all data as parametric, and the comparisons of the continuous variables between the two groups of patients were done using the Student's t-test (unpaired or paired analysis where appropriate). Continuous data are expressed as mean ± standard deviation (SD). The categorical variables were analysed using the chi-square test and expressed as number (percentage). Correlations between the circulating levels of Plg or PAI-1 and IL-6 were calculated by Pearson's coefficients (R). We also performed a binary logistic regression analysis to predict the relationship between Plg or PAI-1 and IL-6 levels and calculated the R^2 value. $P < 0.05$ was considered statistically significant. Outliers were excluded from the analyses.

References

1. Vago JP, Sugimoto MA, Lima KM, Negreiros-Lima GL, Baik N, Teixeira MM, et al. Plasminogen and the Plasminogen Receptor, Plg-RKT, Regulate Macrophage Phenotypic, and Functional Changes. *Front Immunol.* 2019;10:1458.
2. Sugimoto MA, Ribeiro ALC, Costa BRC, Vago JP, Lima KM, Carneiro FS, et al. Plasmin and plasminogen induce macrophage reprogramming and regulate key steps of inflammation resolution via annexin A1. *Blood.* 2017;129(21):2896-907.
3. Carmo AA, Costa BR, Vago JP, de Oliveira LC, Tavares LP, Nogueira CR, et al. Plasmin induces in vivo monocyte recruitment through protease-activated receptor-1-, MEK/ERK-, and CCR2-mediated signaling. *J Immunol.* 2014;193(7):3654-63.
4. Borg RJ, Samson AL, Au AE, Scholzen A, Fuchsberger M, Kong YY, et al. Dendritic Cell-Mediated Phagocytosis but Not Immune Activation Is Enhanced by Plasmin. *PLoS One.* 2015;10(7):e0131216.
5. Rosenwald M, Koppe U, Keppeler H, Sauer G, Hennel R, Ernst A, et al. Serum-derived plasminogen is activated by apoptotic cells and promotes their phagocytic clearance. *J Immunol.* 2012;189(12):5722-8.
6. Draxler DF, Daglas M, Fernando A, Hanafi G, McCutcheon F, Ho H, et al. Tranexamic acid modulates the cellular immune profile after traumatic brain injury in mice without hyperfibrinolysis. *J Thromb Haemost.* 2019;17(12):2174-87.
7. Zaidan I, Tavares LP, Sugimoto MA, Lima KM, Negreiros-Lima GL, Teixeira LC, et al. Angiotensin-(1-7)/MasR axis promotes migration of monocytes/macrophages with a regulatory phenotype to perform phagocytosis and efferocytosis. *JCI Insight.* 2021.
8. Machado MG, Tavares LP, Souza GVS, Queiroz-Junior CM, Ascencao FR, Lopes ME, et al. The Annexin A1/FPR2 pathway controls the inflammatory response and bacterial dissemination in experimental pneumococcal pneumonia. *FASEB J.* 2020;34(2):2749-64.
9. Colon DF, Wanderley CW, Franchin M, Silva CM, Hiroki CH, Castanheira FVS, et al. Neutrophil extracellular traps (NETs) exacerbate severity of infant sepsis. *Crit Care.* 2019;23(1):113.
10. Luo L, Zhang S, Wang Y, Rahman M, Syk I, Zhang E, et al. Proinflammatory role of neutrophil extracellular traps in abdominal sepsis. *Am J Physiol Lung Cell Mol Physiol.* 2014;307(7):L586-96.
11. Silva CMS, Wanderley CWS, Veras FP, Sonego F, Nascimento DC, Goncalves AV, et al. Gasdermin D inhibition prevents multiple organ dysfunction during sepsis by blocking NET formation. *Blood.* 2021;138(25):2702-13.
12. Singer M, Deutschman CS, Seymour CW, Shankar-Hari M, Annane D, Bauer M, et al. The Third International Consensus Definitions for Sepsis and Septic Shock (Sepsis-3). *JAMA.* 2016;315(8):801-10.
13. Seymour CW, Liu VX, Iwashyna TJ, Brunkhorst FM, Rea TD, Scherag A, et al. Assessment of Clinical Criteria for Sepsis: For the Third International Consensus Definitions for Sepsis and Septic Shock (Sepsis-3). *JAMA.* 2016;315(8):762-74.
14. Shankar-Hari M, Phillips GS, Levy ML, Seymour CW, Liu VX, Deutschman CS, et al. Developing a New Definition and Assessing New Clinical Criteria for

Septic Shock: For the Third International Consensus Definitions for Sepsis and Septic Shock (Sepsis-3). *JAMA.* 2016;315(8):775-87.

15. Barcelos LS, Talvani A, Teixeira AS, Vieira LQ, Cassali GD, Andrade SP, et al. Impaired inflammatory angiogenesis, but not leukocyte influx, in mice lacking TNFR1. *J Leukoc Biol.* 2005;78(2):352-8.

Supplementary Figure 1

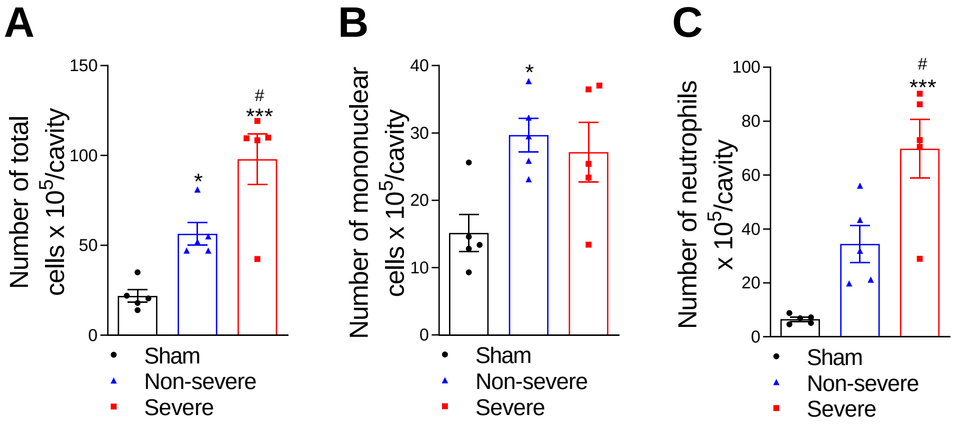

Figure S1: Leukocyte recruitment to the peritoneal cavity in severe and non-severe sepsis. C57BL/6J mice (n=5) were subjected to severe (18G needle) and non-severe (30G needle) CLP. Cells present in the peritoneal cavity were harvested 12 hours after CLP. The number of total cells (A), mononuclear cells (B) and neutrophils (C) were evaluated by counting cytospin slides stained with May-Grunwald-Giemsa. Results are shown as the mean ± SEM of at least five mice per group. *P < 0.05, ***P < 0.001 when comparing sham with CLP group. #P < 0.05 when comparing severe and non-severe sepsis.

Supplementary Figure 2

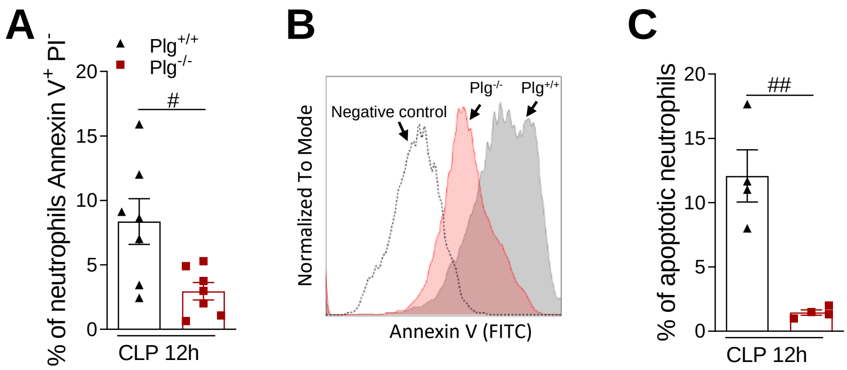

Figure S2: Assessment of apoptosis in Plg$^{-/-}$ mice and their wild type littermates during non-severe sepsis. Plg$^{+/+}$ and Plg$^{-/-}$ mice were subjected to non-severe (30G needle) CLP. Cells present in the peritoneal cavity were harvested 12 hours after CLP. The frequency of annexin V^{+} neutrophils were determined by flow cytometry (A). Representative histograms are shown (B). The percentage of apoptotic neutrophils (C) was evaluated by counting cytospin slides stained with May-Grunwald-Giemsa. $^{\#}$P < 0.05 and $^{\#\#}$P < 0.01 when comparing Plg$^{+/+}$ and Plg$^{-/-}$ mice.

Supplementary Figure 3

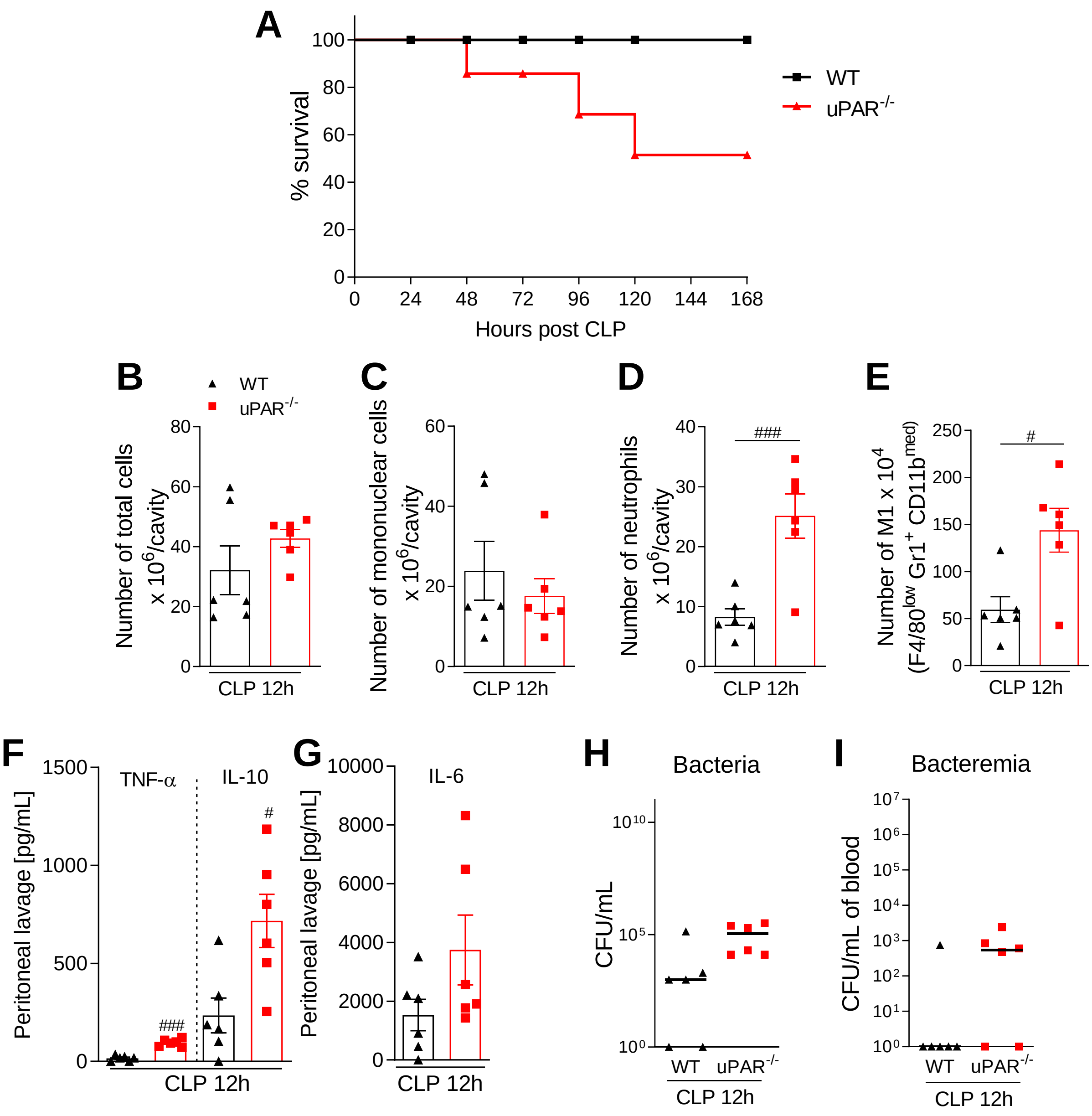

Figure S3: Assessment of survival rates and inflammatory parameters in uPAR$^{-/-}$ mice and their wild type littermates during non-severe sepsis. uPAR$^{+/+}$ and uPAR$^{-/-}$ mice were subjected to non-severe (30G needle) CLP. The survival rates were monitored for 7 days (A). Cells present in the peritoneal cavity were harvested 12 hours after CLP. The number of total cells (B), mononuclear cells (C) and neutrophils (D) were evaluated by counting cytospin slides stained with May-Grunwald-Giemsa. The number of M1 [F4/80^{low} GR1^{+} CD11b^{med}] macrophages were determined by flow cytometry (E). The levels of TNF, IL-10 and IL-6 were quantified in cell-free peritoneal lavages by ELISA (F, G). The peritoneal lavage (H) and blood (I) were plated in BHI media for the analysis of bacterial load. Results are shown as the mean ± SEM of at least five mice per group. $^{\#}P < 0.05$ and $^{\#\#\#}P < 0.001$ when comparing WT and uPAR$^{-/-}$ mice. CFU, colony-forming unit.

Supplementary Figure 4

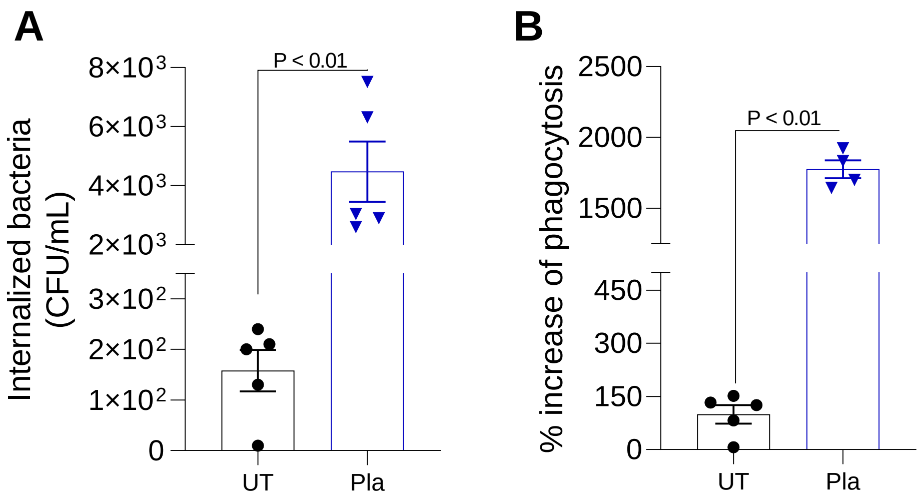

Figure S4: Plasmin increases in vitro macrophage phagocytosis of *Escherichia coli*. Bone marrow derived macrophages (2 × 10^5) were pretreated with Plasmin (4 µg/mL) for 20 hours. Then, cells were incubated with E. coli (MOI 10) for 3 hours to allow adhesion (1h at 4 ^oC) followed by phagocytosis (2h at 37 ^oC). Noninternalized bacteria were excluded by the incubation of the cells with gentamicin, followed by the lysis of macrophages to identify the number of viable phagocytosed bacteria. Results are expressed as CFU/mL of internalized bacteria (A) or % increase of phagocytosis (B) (CFU counts on LB agar plates).

Supplementary Figure 5

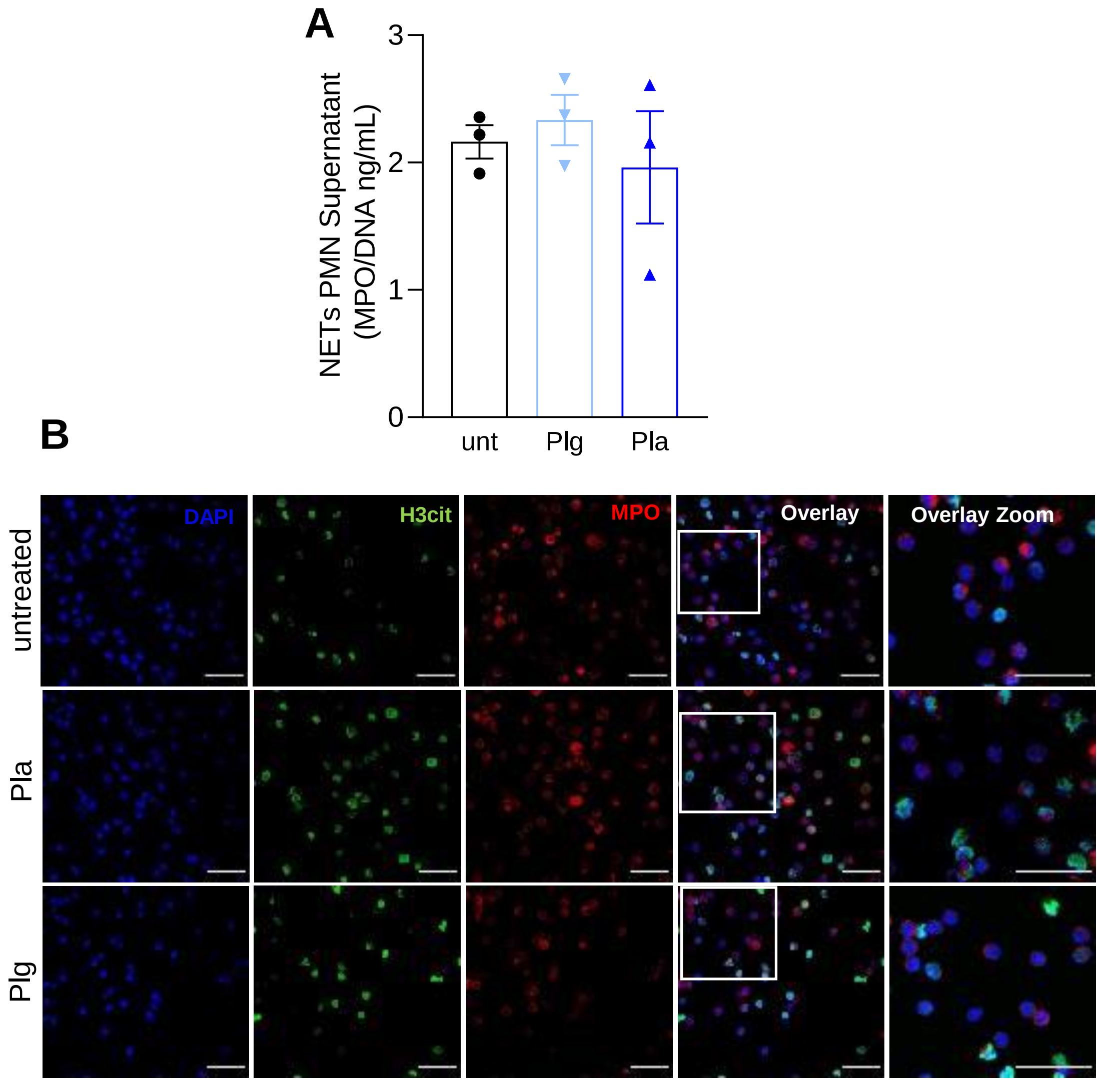

Figure S5: Effects of plasminogen and plasmin on NETs release in vitro. Bone marrow neutrophils obtained from C57BL/6J mice were treated with Plg (4 µg/mL) and Pla (4 µg/mL) for 5h. Quantification of NETs release (MPO/DNA) in supernatant (A). Representative fluorescence images of NETs stained for DNA (DAPI, blue), citrullinated histone H3 (H3cit, green) and myeloperoxidase (MPO, red), are shown (B). The scale bar indicates 50 µm at 630x magnification.

Supplementary Table 1

Table 1. Demographic and clinical parameters of patients with sepsis and septic shock.

		Sepsis (n=14)	Septic shock (n=10)	*P* value
**Age (years-old)**		63 ± 15	61 ± 12	0.666[a]
**Sex**	**Female (n/%)**	10 (71%)	3 (30%)	0.045*[b]
	**Male (n/%)**	4 (29%)	7 (70%)	
**SOFA at admission (points)**		5.0 ± 2.5	9.2 ± 4.4	0.006**[a]
**Total leukocytes (Ref: 3.6-11.2 K/µL)**		11.45 ± 5.08	17.75 ± 8.39	0.0318*[a]
**Neutrophils (Ref: 1.8-7.8 K/µL)**		9.14 ± 3.21	14.14 ± 6.60	0.0218*[a]
**Lactate levels (Ref: 0.1-1.3 mg/dL)**		1.97 ± 0.716	2.82 ± 0.967	0.0252*[a]
**C reactive protein (Ref: ≤ 10 mg/L)**		159.8 ± 130.4	313.9 ± 117.3	0.007**[a]
**Hemoglobin (Ref: 12.5-16.3 mg/dL)**		10.76 ± 2.29	9.84 ± 2.27	0.3391[a]
**Platelets (Ref: 159-386 K/µL)**		237 ± 119	222 ± 142	0.7756[a]

Ref, reference range.; SOFA, Sequential Organ Failure Assessment. *P < 0.05, **P < 0.01 ([a] Student's t-test or [b] chi-square test). Continuous data are expressed as mean ± standard deviation and categorical variables as number (percentage).

Supplementary Table 2

Table 2. Significant correlations between plasminogen levels and laboratory parameters in patients with sepsis and/or septic shock according to hospitalization day.

	**Day 1**		**Day 3**	
**Sepsis + septic shock**	R	*P*	R	*P*
Plg *vs.* platelets	0,635	0,002	NS	NS
Plg *vs.* C reactive protein	NS	NS	-0,525	0,012
**Sepsis**				
Plg *vs.* platelets	0,615	0,033	NS	NS
Plg *vs.* C reactive protein	-0,574	0,051	NS	NS
**Septic shock**				
Plg *vs.* platelets	0,656	0,040	NS	NS

Plg, plasminogen; R, Pearson's correlation coefficient; NS, non-significant correlation.

Supplementary Table 3

Table 3. Plg$^{+/+}$ and Plg$^{-/-}$ mice numbers of leukocytes in blood after CLP

	**Plg$^{+/+}$**	**Plg$^{-/-}$**	***P* value**
**Total cells (K/ul)**	3.96 ± 2.88	11.15 ± 9.29	*P* = 0.0236*
**Lymphocytes (K/ul)**	1.19 ± 1.30	2.68 ± 2.23	*P* = 0.0624
**Monocytes (K/ul)**	1.20 ± 2.29	2.32 ± 3.18	*P* = 0.3557
**Neutrophils (K/ul)**	2.31 ± 1.65	5.81 ± 5.08	*P* = 0.0425*
**Platelets (K/ul)**	289 ± 204	313 ± 145	*P* = 0.7532

t test $*P < 0.05$
